# Development and Validation of Liquid Chromatographic Method for Fast Determination of Lincomycin, Polymyxin and Vancomycin in Preservation Solution for Transplants

**DOI:** 10.3390/molecules29133166

**Published:** 2024-07-03

**Authors:** Qi Lin, Tam Nguyen, Chiara Staffieri, Ann Van Schepdael, Erwin Adams

**Affiliations:** Department of Pharmaceutical and Pharmacological Sciences, Pharmaceutical Analysis, KU Leuven, Herestraat 49, O&N2, PB 923, 3000 Leuven, Belgium; qi.lin923@gmail.com (Q.L.); thiminhtam.nguyen@kuleuven.be (T.N.); staffieri.chiara@gmail.com (C.S.); ann.vanschepdael@kuleuven.be (A.V.S.)

**Keywords:** transplant preservation solution, lincomycin, polymyxin, vancomycin, superficially porous particles, liquid chromatography, validation

## Abstract

In this study, a liquid chromatographic method was developed for the fast determination of lincomycin, polymyxin and vancomycin in a preservation solution for transplants. A Kinetex EVO C18 (150 × 4.6 mm, 2.6 µm) column was utilized at 45 °C. Gradient elution was applied using a mixture of mobile phases A and B, both including 30 mM phosphate buffer at pH 2.0 and acetonitrile, at a ratio of 95:5 (*v*/*v*) for A and 50:50 (*v*/*v*) for B. A flow rate of 1.0 mL/min, an injection volume of 20 µL and UV detection at 210 nm were used. A degradation study treating the three antibiotics with 0.5 M hydrochloric acid, 0.5 M sodium hydroxide and 3% H_2_O_2_ indicated that the developed method was selective toward lincomycin, polymyxin, vancomycin and their degradation products. Other ingredients of the preservation solution, like those from the cell culture medium, did not interfere. The method was validated with good sensitivity, linearity, precision and accuracy. Furthermore, lincomycin, polymyxin and vancomycin were found to be stable in this preservation solution for 4 weeks when stored at −20 °C.

## 1. Introduction

Antibiotics are added to solutions to preserve transplants for different reasons, for example, to prevent bacterial growth before and immediately after transplantation [1,2], to promote the proliferation of transplanted cells [3] and to instruct and develop the immune system of the recipients [4]. Lincomycin is a lincosamide used for treating severe bacterial infections. It has a narrow antibiotic spectrum showing antibacterial activity against Gram-positive and pathogenic species like *Streptococcus*, *Staphylococcus* and *Mycoplasma* [4]. Polymyxin is a biosynthesized complex consisting of 10 amino acid residuals which is mainly active against Gram-negative bacteria [5]. Vancomycin is generated from the *Amycolatopsis orientalis* strain. It is suggested to be used after the failure of other antibiotics or when treating serious or life-threatening infections [6].

A preservation solution containing these three antibiotics in a matrix of RPMI 1640 Medium (which includes inorganic salts, amino acids, vitamins, nutrients like glucose, pH indicator, etc.) is generally prepared by hospitals involved in organ transplants. However, the solution should be controlled before use to check if it was properly prepared and that the assay values comply with the limits. Moreover, the stability of the antibiotics in this preservation solution is not clear; it is not known for how long and under which conditions it can best be stored. Hence, a sensitive and reliable analytical method will be helpful to determine the three antibiotics. 

Liquid chromatography (LC), as an efficient analytical tool to separate complex samples, has been employed. Several LC methods have been described for the analysis of lincomycin, polymyxin and vancomycin separately, but not in a mixture of all three. For example, the LC separation of lincomycin from its related substances has been described by Orwa et al. [7]. The separation of vancomycin from its impurities was published by Diana et al. [8]. In addition, Mathew and Das Gupta [9] and Orwa et al. [10] dedicated two LC methods to assess the stability of vancomycin and polymyxin, respectively. The European Pharmacopoeia (Ph. Eur.) also adopted LC methods in the respective monographs of the three antibiotics [11]. Only Cheng et al. [12] published an LC-MS method for the determination of polymyxin and vancomycin in rat plasma. However, after checking, their method did not chromatographically separate polymyxin and vancomycin. 

Since no chromatographic method has been described in the literature to separate lincomycin, polymyxin and vancomycin in a single run, the aim of this study was to develop and validate an LC-UV method for the determination of the three antibiotics simultaneously. The separation was complicated by RPMI 1640 Medium, which contains numerous compounds that are also present in the sample. The method should also be able to evaluate the stability of the preservation solution. Although lincomycin and polymyxin are mixtures of active compounds, no efforts will be made to separate all of these components, but they will be analyzed as the respective cluster if they are not separated.

## 2. Results and Discussion

### 2.1. Method Development

In this study, an LC-UV method was developed to determine lincomycin hydrochloride (120 µg/mL), polymyxin sulfate (100 µg/mL) and vancomycin hydrochloride (50 µg/mL) in an RPMI 1640 matrix used as a preservation solution for transplants. The three antibiotics were combined to obtain antibacterial activity against a broad range of micro-organisms. Since it is the intention to use the method routinely in quality control laboratories, LC-UV was preferred over LC-MS because of its robustness and lower operational and purchase costs.

First, a comparison was made of the LC methods prescribed in the related substance test of the respective monographs in the Ph. Eur. [11] to set the starting point of the LC conditions on the Kinetex EVO C18 (150 × 4.6 mm, 2.6 µm) column. It can be derived from the overview in Table 1 that for simultaneous analyses of the three antibiotics, the following conditions should be beneficial: (1) a reversed-phase column, preferably end-capped and base-deactivated, which is the case for the Kinetex EVO C18 (150 × 4.6 mm, 2.6 µm) column; (2) a moderate column temperature avoiding on-column degradation of the analytes; (3) a mobile phase containing acetonitrile (ACN) as an organic modifier and a buffer solution with a pH between 2 and 7; (4) a flow rate of 1 mL/min; (5) UV detection at low wavelengths since lincomycin and polymyxin have no strong UV absorbing chromophore. 

The initial column temperature was set at 30 °C. The mobile phase was a gradient mixture from solution A that was composed of 90 volumes of phosphate buffer (30 mM, pH 3.0) and 10 volumes of ACN, and solution B that consisted of the same components, but at a ratio of 50:50, *v*/*v*. A flow rate of 1.0 mL/min and a UV detection wavelength at 210 nm were selected. Further optimization was implemented for the pH, concentration of phosphate buffer, amount of ACN in solution A, column temperature and UV wavelength.

The influence of buffer pH was investigated in the range from pH 2.0 to 7.0. It was observed that the retention of lincomycin was higher with increasing buffer pH across the range examined. For vancomycin, its retention showed a U-shaped relationship with pH. The retention reached a minimum at pH 5.0. Furthermore, a reversed U-shaped relationship was found for buffer pH and the retention of polymyxin, which showed maximum peak retention at pH 5.0. In addition, the UV response of polymyxin became weaker and no good peak shape could be obtained when buffers above pH 5.0 were used. Moreover, in a stability study published by Orwa et al. [10], it is mentioned that the degradation of polymyxin easily happened in solutions with pH values above 5 and that it is more stable in acidic media. Concerning vancomycin, it has been reported by Mathew and Das Gupta [9] that higher concentrations of phosphate ions accelerate the degradation of the antibiotic and that this is more pronounced at about pH 7 than pH 4. So, a buffer with pH 2.0 was selected, since this ensured the buffer capacity of phosphate and led to the best resolution between lincomycin, polymyxin and vancomycin, as well as the components of RPMI 1640 Medium.

The performance of different concentrations of phosphate buffer was assessed at 20 and 30 mM. Vancomycin and polymyxin showed a better peak shape (peak symmetry ≤ 1.5) when the higher concentration was used. The concentration was not further increased in order to not compromise the stability of vancomycin. The shape of the lincomycin peak was not influenced by either concentration. The retention times of the three antibiotics hardly changed between using 20 and 30 mM of phosphate buffer. So, 30 mM of buffer was preferred. 

Reducing the amount of ACN from 10% to 5% in solution A resulted in an increase in retention for lincomycin, polymyxin and vancomycin. Vancomycin was the most affected, followed by lincomycin and then polymyxin. 

The column temperature had an effect on the retention times as could be expected. Among the investigated temperatures from 30 °C to 55 °C, shorter retention times of several minutes were found with raising column temperature. The best separation of the three antibiotics and components of RPMI 1640 Medium within a reasonable analysis time was observed at a column temperature of 45 °C. 

Next, UV detection at 205 nm, 210 nm, 215 nm and 220 nm was evaluated concerning the response intensity versus the noise. The most intense response was found at 205 nm, and it was reduced with increasing wavelengths. However, the lowest wavelength also showed the highest baseline noise. The best signal-to-noise ratio and baseline stability were obtained at 210 nm; so, this wavelength was selected for the final method. 

Furthermore, it was observed that regarding the preparation of the reference solution, polymyxin was more stable when it was dissolved in the mobile phase than in water, while lincomycin and vancomycin were stable in both solvents. For the sample solution, no diluent was chosen since the preservation solution was injected as such, owing to the low concentration of the three antibiotics. It was also well buffered by RPMI 1640 Medium. 

**Figure 1:**
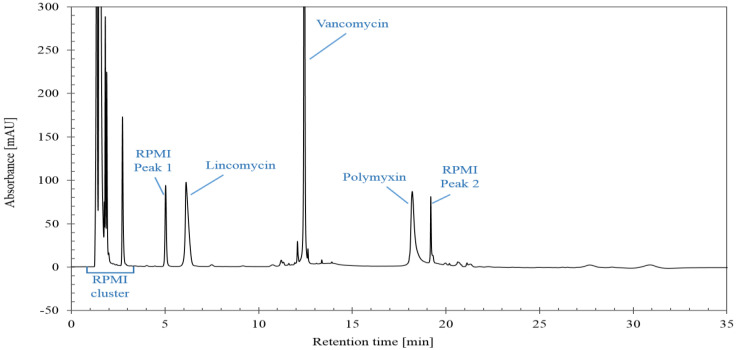
Chromatogram obtained with optimized LC-UV conditions for mixture consisting of lincomycin, polymyxin, vancomycin and RPMI Medium 1640.

A chromatogram obtained with the optimized LC-UV method is illustrated in Figure 1. Both lincomycin and polymyxin are each eluted as a single peak and not as a cluster of peaks corresponding to the different compounds. This simplifies the integration and is sufficient to check the total content of the three antibiotics.

### 2.2. Method Validation

#### 2.2.1. Selectivity

**Figure 2:**
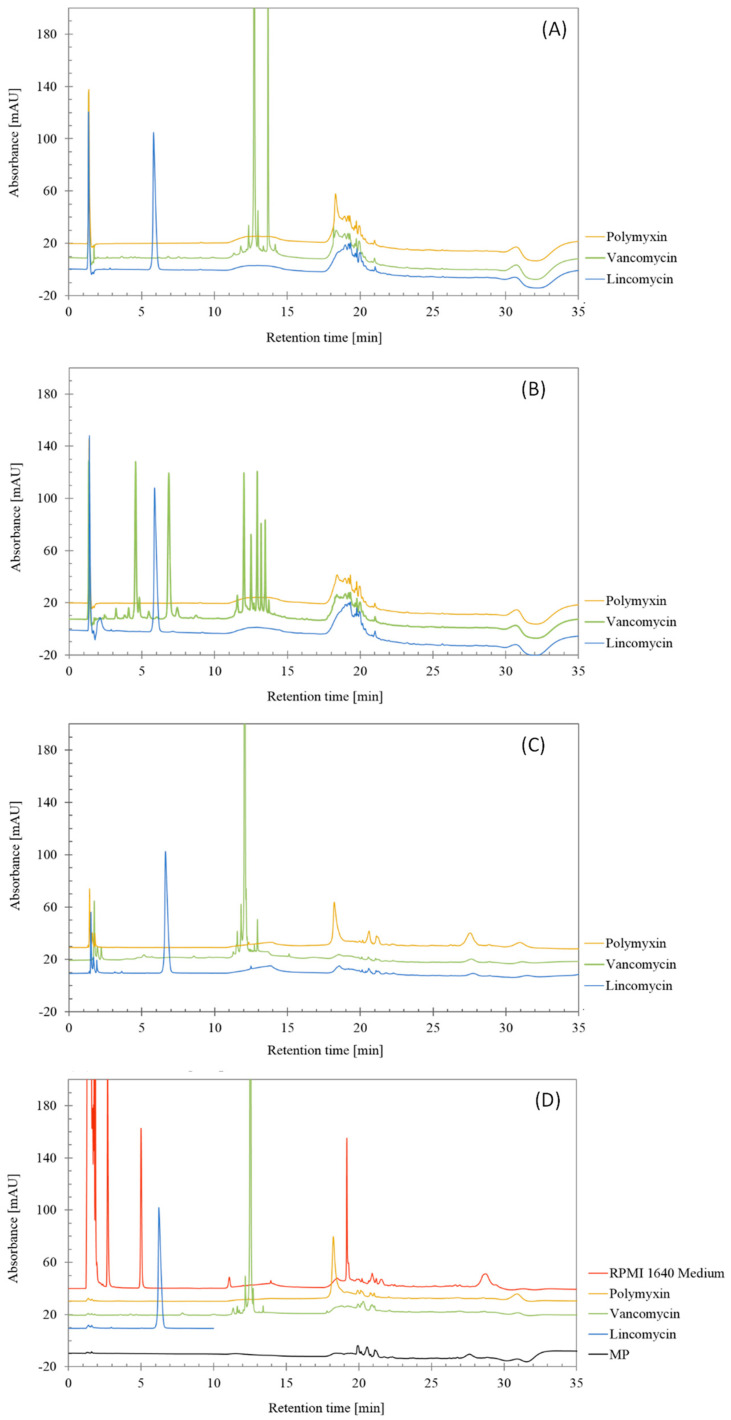
Chromatograms obtained from forced degradation studies of lincomycin, polymyxin and vancomycin treated by (**A**) 0.5 M HCl, (**B**) 0.5 M NaOH, (**C**) 3% H_2_O_2_ and (**D**) non-treated antibiotics, RPMI 1640 Medium and mobile phase (MP).

From the chromatograms shown in Figure 2 that are corresponding to injections of degraded lincomycin, polymyxin and vancomycin after three ways of forced degradation, the RPMI Medium 1640 and the mobile phase, it can be derived that no interferences were found in the main peaks of lincomycin and vancomycin. 

#### 2.2.2. Sensitivity

The limit of detection (LOD) of lincomycin, polymyxin and vancomycin was found to be 24 ng, 24 ng and 3 ng, respectively, expressed as the amount injected on the column corresponding to a signal-to-noise ratio of 3. Amounts of 80 ng for lincomycin, 80 ng for polymyxin and 10 ng for vancomycin were demonstrated to be the limit of quantification (LOQ), determined at a signal-to-noise ratio of 10.

#### 2.2.3. Linearity

Three regression equations were calculated as follows: *y* = 0.1694 *x* + 0.1100 (R^2^ = 0.9998) for lincomycin; *y* = 0.2280 *x* − 0.1391 (R^2^ = 0.9995) for polymyxin; and *y* = 1.4617 *x* + 0.0946 (R^2^ > 0.9999) for vancomycin. So, the R^2^ values were clearly above the acceptance value of 0.995. Further, it was confirmed that zero was included in the 95% confidence interval (CI) of the y intercepts in all three equations, and their residual plots were randomly distributed. In addition, a lack-of-fit test for the linearity model revealed *p*-values above 0.05 for lincomycin, polymyxin and vancomycin. It may be concluded that linearity was established for the three antibiotics in the examined range.

#### 2.2.4. Accuracy

Good accuracy of this method was demonstrated by the recoveries, which were each determined in triplicate and amounted at the 80, 100 and 120% level to, respectively, 101.9% (RSD: 0.5%), 101.8% (RSD: 0.5%) and 101.6% (RSD: 0.7%) for lincomycin; 99.9% (RSD: 0.6%), 99.8% (RSD: 1.2%) and 98.5% (RSD: 1.3%) for polymyxin; and 99.8% (RSD: 0.2%), 99.1% (RSD: 0.4%) and 100.1% (RSD: 0.3%) for vancomycin. All of these values fell within the acceptance range of 98–102%.

#### 2.2.5. Precision

The precision results were expressed as relative standard deviation (RSD). The intra-day precision found on each day was not more than 1.4% for lincomycin, 1.2% for polymyxin and 1.0% for vancomycin. The inter-day precision investigated over 18 injections in three days was 1.2%, 1.5% and 1.2% for lincomycin, polymyxin and vancomycin, respectively. Since none of the values exceeded 2%, the precision was found to be acceptable.

### 2.3. Stability of Sample Solution 

At release (0 h), the three antibiotics complied with the 95–105% limits. More detailed results are shown in Table 2. After 4 weeks, the content of lincomycin and polymyxin remained within these limits under both storage conditions. However, the vancomycin content was observed to decrease. When stored at −20 °C for 4 weeks, around 93% of vancomycin was left, which is still within the end of the shelf-life specifications (90–110%). When stored at 4 °C, the sample could only be used for up to 2 weeks. 

## 3. Materials and Methods

### 3.1. Chemicals 

ACN (HPLC grade) was purchased from Fisher Scientific (Leicestershire, UK). Dipotassium hydrogen phosphate was provided by Acros Organics (Geel, Belgium). Potassium dihydrogen phosphate was obtained from Merck Millipore (Darmstadt, Germany) and 85% (*w/w*) phosphoric acid from Chem-Lab (Zedelgem, Belgium). RPMI 1640 Medium was obtained from Thermo Fisher (Paisley, UK). A Milli-Q water purification system from Millipore (Bedford, MA, USA) produced the purified water used in this study. 

Lincomycin hydrochloride and polymyxin sulfate from EDQM (Strasbourg, France) and vancomycin hydrochloride from Xellia (Copenhagen, Denmark) that were used to prepare the preservation solution were also used as reference substances.

### 3.2. Instrumentation and Chromatographic Conditions

The LC-UV system used for method development and validation consisted of an LPG-3400A pump and an ASI-100 autosampler from Dionex (Sunnyvale, CA, USA), and a Spectra LINEAR UVIS 200 detector from Thermo Separation Products (San Jose, CA, USA). Chromeleon 6.8 software from Dionex was utilized for LC system operation and data acquisition. A Julabo ED heating thermostat (Seelbach, Germany) was immersed in a water bath to maintain the column temperature.

The separation was performed on a Kinetex EVO C18 (150 × 4.6 mm, 2.6 µm) column from Phenomenex (Torrance, CA, USA). The final mobile phase was a gradient mixture of mobile phases A and B that were both composed of ACN and 30 mM phosphate buffer with pH 2.0, but with different composition ratios, 5:95 (*v*/*v*) for A and 50:50 (*v*/*v*) for B. The gradient started with 100% of mobile phase A and remained the same for 8 min. In the next 3 min, mobile phase B increased linearly to 15%. This ratio was kept for 4 min before mobile phase B quickly reached 100% in 2 min. The mobile phase was isocratic for the next 10 min and took 1.5 min to return to the initial composition followed by the re-equilibration time. The flow rate was 1.0 mL/min, the column temperature was 45 °C, the injection volume was 20 µL and the detection wavelength was 210 nm.

### 3.3. Solution Preparation

The preservation solution was prepared by adding 100 µL of 300 mg/mL lincomycin hydrochloride, 250 µL of 100 mg/mL polymyxin sulfate, and 250 µL of 50 mg/mL vancomycin hydrochloride, all in water, to 250 mL of RPMI 1640 Medium. So, the solution consisted of 120 µg/mL of lincomycin hydrochloride, 100 µg/mL of polymyxin sulfate and 50 µg/mL of vancomycin hydrochloride in the matrix. It was injected as such. 

Reference solutions were prepared at the same concentrations as the sample solution using mobile phase A. For the analysis of samples and validation of the linearity, the reference solution was prepared as a mixture of the 3 antibiotics. For verification of the accuracy, the antibiotics were injected separately.

### 3.4. Validation

The developed method was validated in accordance with the ICH Q2 guideline [13], including selectivity, sensitivity, linearity, precision and accuracy, as described below.

- Selectivity

The selectivity of the method was assessed by injecting the blank solution (mobile phase A), the placebo matrix solution (RPMI 1640 solution) and the forced degradation solutions in order to verify the selectivity among the major compounds, degradation products and RPMI 1640 components. The forced degradation solutions were prepared by separately treating lincomycin, polymyxin and vancomycin with 0.5 M HCl for 6 h at ambient temperature, 0.5 M NaOH for 6 h at ambient temperature and 3% H_2_O_2_ for 6 h at 60 °C. The chromatograms were examined to confirm that there was no interference at the retention time of the active compounds.

- Sensitivity

The LOD and LOQ were evaluated by separately injecting the solutions of each antibiotic, which were diluted until the achievement of signal-to-noise ratios of 3 and 10, respectively.

- Linearity

The calibration curves, implying the relationship between sample concentration (*x*) and UV response (*y*), were evaluated for lincomycin, polymyxin and vancomycin in the ranges from 30 to 150 µg/mL, from 25 to 125 µg/mL, and from 12.5 to 62.5 µg/mL, respectively. The UV signals generated from five concentrations were determined in triplicate for each antibiotic. The slope, intercept and determination coefficient (R^2^) from the calibration curve were determined. R^2^ should not be less than 0.995. The residual plots should be randomly distributed with zero included in the 95% confidence interval (CI) of the y intercepts.

- Accuracy

Since the sample was a preservation solution consisting of lincomycin, polymyxin, vancomycin and RPMI 1640 Medium, a comparison of responses measured from an RPMI 1640 solution spiked with the individual drug substances was made to assess the recovery of each antibiotic when using the developed method. The solutions were prepared with known amounts of lincomycin, polymyxin and vancomycin at concentrations of 80%, 100% and 120% of these in the sample, namely 96, 120 and 144 µg/mL, 80, 100 and 120 µg/mL, and 40, 50 and 60 µg/mL, respectively. Corresponding amounts of the three drug components were added to RPMI 1640 Medium to reach concentrations from 80% to 120%, as prescribed in the preservation solution. The solutions were injected in triplicate and the recovery at each concentration level was calculated. The recovery values should be between 98% and 102%.

- Precision

Both intra- and inter-day precisions were examined using the UV responses obtained for 120 µg/mL of lincomycin, 100 µg/mL of polymyxin and 50 µg/mL of vancomycin. Six consecutive injections were measured at each of the three days. RSD values of less than 2% should be obtained.

### 3.5. Stability of Sample Solution

The stability of the preservation solution was evaluated for 4 weeks after preparation and stored at 4 °C in a refrigerator and at −20 °C in a freezer. The preservation solution was injected in triplicate immediately after preparation (0 h) and at 1 week, 2 weeks, 3 weeks and 4 weeks. Reference solutions were freshly prepared at each time point. 

## 4. Conclusions

An LC-UV method for the simultaneous determination of lincomycin, polymyxin and vancomycin, in the presence of a complex matrix, was developed on a Kinetex EVO C18 column. This is the first time that a method has been described to separate those three antibiotics. Since no sample pretreatment is necessary and a conventional LC-UV system can be used, the method is simple, which is an advantage when applied in quality control laboratories. Good validation data were obtained and demonstrated that this method is selective, sensitive, linear, precise and accurate. This method is useful for quality control of the content and stability of the three antibiotics in the preservation solution. At the concentrations studied, the solution was stable for up to 4 weeks at −20 °C. However, if the solution was kept at 4 °C, a stability of 2 weeks should be taken into account.

## Figures and Tables

**Table 1:** An overview of the LC methods prescribed in the related substance test of the respective monographs in the Ph. Eur. [11].

	Lincomycin Hydrochloride	Polymyxin B Sulfate	Vancomycin Hydrochloride
Stationary phase	End-capped base-deactivated octylsilyl silica gel (250 × 4.6 mm, 5 µm)	End-capped octadecylsilyl silica gel (250 × 4.6 mm, 5 µm)	Octadecylsilyl silica gel (250 × 4.6 mm, 5 µm)
Column temperature	50 °C	30 °C	Room temperature
Mobile phase	MeOH-ACN–phosphate buffer (0.15 M, pH 6.1), 8:17:75 (*v*/*v*/*v*)	ACN—4.46 g/L of Na_2_SO_4_ (to pH 2.3 with 31.4 mM H_3_PO_4_ solution), 20:80 (*v*/*v*)	4 mL/L of Triethylamine (to pH 3.2 with H_3_PO_4_ solution)-THF-ACN, gradient with A) 920:10:70 (*v*/*v*/*v*) and B) 700:10:290 (*v*/*v*/*v*)
Flow rate	1.0 mL/min	1.0 mL/min	1.0 mL/min
UV detection	210 nm	215 nm	280 nm

**Table 2:** Contents (RSD, n = 3) of lincomycin, polymyxin and vancomycin at different durations and conditions of storage.

	Lincomycin		Polymyxin		Vancomycin	
	4 °C	−20 °C	4 °C	−20 °C	4 °C	−20 °C
0 h	101.8% (0.5%)		99.4% (1.2%)		99.6% (1.3%)	
1 week	99.7% (0.6%)	99.6% (1.0%)	99.4% (1.6%)	101.0% (0.6%)	95.2% (1.8%)	97.9% (2.0%)
2 weeks	98.3% (0.2%)	97.6% (0.2%)	98.6% (1.6%)	99.6% (0.2%)	92.8% (0.1%)	96.1% (0.2%)
3 weeks	98.2% (0.3%)	98.5% (0.3%)	97.5% (1.4%)	101.8% (0.7%)	88.1% (0.2%)	93.5% (0.2%)
4 weeks	99.4% (0.2%)	98.3% (0.1%)	99.4% (1.7%)	101.0% (1.0%)	86.4% (0.2%)	93.3% (0.4%)

## Data Availability

According to the policy of KU Leuven, the authors may be contacted for more details concerning the data supporting the reported results.
